# Cutaneous human papillomavirus E6 impairs the cGAS-STING pathway

**DOI:** 10.1128/msphere.00859-25

**Published:** 2026-02-26

**Authors:** Grant Brooke, Dalton Dacus, Rose Pollina, Katsura Asano, Nicholas A. Wallace

**Affiliations:** 1 Division of Biology, Kansas State University 5308, Manhattan, Kansas, USA; 2 Department of Kinesiology, Kansas State University 5308, Manhattan, Kansas, USA; Virginia Commonwealth University, Richmond, Virginia, USA

**Keywords:** human papillomavirus, innate immunity, cGAS-STING, β-HPV

## Abstract

Beta human papillomaviruses (β-HPVs) are ubiquitous double-stranded DNA (dsDNA) viruses that may promote skin cancers by destabilizing the host genome. Supporting this, expression of the E6 gene from a β-HPV (β-HPV 8 E6) results in increased micronuclei that should induce an innate immune response that eliminates these cells. However, β-HPV 8 E6 promotes rather than restricts proliferation. We hypothesize that β-HPV 8 E6 accomplishes this by attenuating the cyclic GMP-AMP synthase-stimulator of interferon genes (cGAS-STING) pathway, an innate immune pathway that becomes activated in response to cytosolic dsDNA. Here, we show that in response to dsDNA transfection, β-HPV 8 E6 reduced the intensity of cGAS-STING pathway activation via a reduction in STING phosphorylation. Additionally, our unbiased assessment found that β-HPV 8 E6 broadly downregulates innate immunity. This impairment of the cGAS-STING innate immune response could contribute to the prevalence of β-HPV infections.

**IMPORTANCE:**

Beta human papillomaviruses (β-HPVs) may promote non-melanoma skin cancers in certain immunocompromised populations by destabilizing the host genome. Our group has previously documented the ability of a specific β-HPV, type 8, to promote proliferation despite antiproliferative stimuli, including mitotic errors such as anaphase bridges, micronuclei, and chromothripsis. The mechanisms β-HPV uses to overcome these challenges are not yet fully elucidated. This paper addresses one possible mechanism by proposing that β-HPVs suppress cGAS-STING signaling to promote proliferation under conditions that would typically initiate an innate immune response, resulting in apoptosis or senescence. We found that β-HPVs can impair cGAS-STING signaling at a post-translational modification level and play a role in broadly downregulating innate immune-associated genes. Similarly, alpha human papillomaviruses (α-HPVs) display the ability to downregulate many of the same interferon-inducible genes. This suggests that there is a shared need between β-HPV and α-HPVs to target innate immune responses.

## INTRODUCTION

Human papillomaviruses (HPVs) are a family of small, non-enveloped double-stranded DNA (dsDNA) viruses divided into five subgenera (alpha-, beta-, gamma-, mu-, and nu-papillomaviruses) based on sequencing homology (1). Of the five genera, alpha human papillomaviruses (α-HPVs) and beta human papillomaviruses (β-HPVs) have been most closely linked with cancer development (2–4). The role of high-risk α-HPVs in tumorigenesis is well established. These viruses primarily cause cancers in the oropharyngeal and anogenital tracts (5–7) through the continued expression of viral oncogenes E6 and E7 (8). β-HPVs are less strongly associated with cancer, apart from a report linking β-HPV genome integration to skin carcinogenesis (9).

β-HPVs are ubiquitous viruses that are commonly spread through skin-to-skin contact (10). Infections often first occur in early childhood with common reinfections (10, 11). While β-HPV infections occur frequently, they are generally transient in nature. Infections typically only last between 8 and 11 months (12). These infections are asymptomatic in immunocompetent people, but a subset of β-HPVs, including β-HPV 8, has been shown to increase the risk of skin cancer in people with the rare genetic disorder epidermodysplasia verruciformis and other forms of immunosuppression (13–17). Mechanistic evidence suggests that the E6 protein from these β-HPVs contributes to this increased risk by altering DNA repair mechanisms and creating genomic instability (18, 19). The result of these alterations includes the increased frequency of chromothripsis, anaphase bridges, and other mitotic errors (20).

In α-HPVs, E6 has also been implicated in impairing innate immune responses triggered by pattern recognition receptors (PRRs) in response to pathogen-associated molecular patterns, including the Toll-like receptors (TLRs), RIG-1-like receptors, and cyclic GMP-AMP-synthase-stimulator of interferon genes (cGAS-STING) (21–29). The cGAS-STING pathway recognizes dsDNA in the cytosol of cells, such as β-HPVs and micronuclei induced by β-HPV E6 (30, 31). Once cGAS detects and binds to cytoplasmic DNA, it synthesizes the secondary messenger cGAMP (cyclic GMP-AMP). cGAMP then migrates to the endoplasmic reticulum to activate STING (stimulator of interferon genes) (31). Once activated, STING translocates to the Golgi apparatus, where TBK1 (tank binding kinase) is recruited. TBK1 autophosphorylates itself and phosphorylates STING. Phosphorylated STING then recruits IRF3, a transcription factor, to be phosphorylated by phosphorylated TBK1 (32). Phosphorylated IRF3 translocates to the nucleus to activate the expression of type 1 interferons (31). Type 1 interferon induction triggers activation of the JAK-STAT pathway, which in turn induces the expression of interferon-stimulated genes (ISGs). This expression will culminate in an apoptotic or senescent response (33, 34).

While α-HPVs have established mechanisms to antagonize PRRs (35–39), the extent to which β-HPVs do the same is unclear. Here, we use immunoblotting and RNA-sequencing approaches to show that β-HPV 8 E6 impairs the cGAS-STING innate immune response. We show that β-HPV 8 E6 reduces ISG production and attenuates the innate immune response, despite cGAS properly localizing to micronuclei, and this is associated with a reduction in STING phosphorylation. Taken together, these data are consistent with the hypothesis that the prevalence of β-HPV 8 is in part due to the ability of β-HPV8 E6 to attenuate the cGAS-STING pathway.

## MATERIALS AND METHODS

### Cell culture

Telomerase-immortalized human foreskin keratinocytes (hTERT HFKs) were provided by Michael Underbrink (University of Texas Medical Branch). Cells were grown in Keratinocyte Growth Medium 2 (PromoCell) supplemented with calcium chloride (PromoCell), SupplementMix (PromoCell), and penicillin-streptomycin (Caisson). hTERT HFKs expressed empty vector control (LXSN) and HA-tagged β-HPV8 E6 driven by the promoter in the LXSN vector.

### Transfections

For immunoblots, hTERT HFK cells were plated in 9 mL of complete growth medium in a 10 cm plate. Cells were used at 70%–80% confluency. In time series transfections, 0.5 µg of pLVX-GFP plasmid was diluted in 200 μL Xfect transfection reagent (631,317, Takara). In DNA dose response transfections, cells were transfected with selected plasmid concentrations 0 µg, 0.25 µg, 0.5 µg, 1 µg, 2.5 µg, and 5 µg diluted in 200 μL Xfect transfection reagent (631,317, Takara). The resulting transfection mixture was incubated at room temperature for 15 min, and then the transfection mixture was added to each plate in a drop-wise manner. Time series plates were incubated for selected time intervals (0 h, 4 h, 12 h, 20 h, 28 h, 36 h, and 48 h) at 37°C. DNA dose response plates were incubated for 20 h at 37°C. Cells were then harvested for immunoblotting.

For RNA sequencing, hTERT HFK cells were plated in 20 mL of complete growth medium in a 15 cm plate. Cells were used at 70%–80% confluency. Cells were transfected with 1 µg of plasmid diluted in 1,200 μL Xfect transfection reagent (631,317, Takara). The mixture was incubated at room temperature for 15 min. The transfection mixture was added to each plate dropwise and incubated for 20 h at 37°C. Cells were harvested for RNA extraction and sequencing.

### Immunoblotting

Cells were washed with ice-cold phosphate-buffered saline (PBS), then lysed with radioimmunoprecipitation assay lysis buffer (Santa Cruz Biotechnology) supplemented with phosphatase inhibitor cocktail 2 (Sigma-Aldrich) and protease inhibitor cocktail (Bimake). Protein concentration was determined with a Pierce bicinchoninic acid protein assay kit (Thermo Scientific). Lysates were run on Novex 4%–12% Tris-glycine WedgeWell mini gels (Invitrogen) and transferred to Immobilon-P membranes (Millipore). Membranes were probed with the following primary antibodies: STING (13647S; Cell Signaling Technology), pSTING (Ser366; catalog no. 19781S; Cell Signaling Technology), VIPERIN (MABF106; Sigma-Aldrich), and C23 (MS-3) HRP (sc-8031; Santa Cruz Biotechnology). After exposure to the matching appropriate horseradish peroxidase-conjugated secondary antibody, membranes were visualized using SuperSignal West Femto maximum sensitivity substrate (Thermo Scientific). Densitometry quantification was performed using ImageJ (NIH, Rockville, MD, USA).

### Immunofluorescent microscopy

Cells were seeded into six-well plates with etched coverslips and grown overnight. Cells were fixed with 4% formaldehyde. Then, 0.1% Triton-X solution in PBS was used to permeabilize the cells, followed by blocking with 3% bovine serum albumin in PBS for 30 min. Cells were then incubated with the following: cGAS (D1D3G; Cell Signaling Technologies) and Lamin B1 (C-12; sc-365214; Santa Cruz Biotechnology). The cells were washed and stained with the following appropriate secondary antibodies: Alexa Fluor 594-goat anti-rabbit (Thermo Scientific, catalog no. A11012) and Alexa Fluor 488 goat anti-mouse (Thermo Scientific, catalog no. A11001). After washing, the cells were stained with 2 μM 4′,6-diamidino-2-phenylindole in PBS and visualized with a Zeiss LSM 770 or a Zeiss LSM 880 Airyscan microscope. Laser power, gain, and the pinhole remained the same between experiments to ensure accurate quantification. Background fluorescence was used as an internal control between samples. Images were analyzed using ImageJ techniques, as described previously (40).

### RNA isolation and purification

Cells were washed with phosphate-buffered saline and then lysed with Trizol reagent. RNA was then isolated using the Zymo Clean and Concentrator Kit (Zymo Research). Sample purity was assessed using the nanodrop software. Samples were then delivered to the Molecular and Cell Biology Core (NIH COBRE, Center on Emerging and Zoonotic Infectious Diseases) at Kansas State University for sequencing.

### RNA sequencing

Two microliters of each sample was used for quantification using Qubit RNA BR Assay Kit (Thermo Fisher Scientific) according to the manufacturer’s instructions in the Qubit fluorometer 4 (Thermo Fisher Scientific). After quantification, all samples were normalized to 40 ng/µL (total 1,000 ng). The samples were also analyzed using Tapestation 4150 (Agilent) using RNA ScreenTape Analysis (Agilent) following manufacturer’s instructions. All samples had RIN (RNA Integrity Number) above 9.4. The library preparation was performed using the kit Illumina Stranded mRNA Prep (Illumina) according to the manufacturer’s instructions. Briefly, the messenger RNAs (mRNAs) are purified using oligo(dT) magnetic beads. Then, the mRNAs are used as templates to synthesize the first-strand cDNA followed by the synthesis of the second strand cDNA. After the double-stranded cDNA synthesis, the samples were submitted to a reaction to add adenine nucleotide to the 3′ ends, here called anchors, to the blunt fragments to prevent the fragments from ligating to each other during the adaptor ligation step. The next step was the library amplification, which specifically amplifies anchor-ligated DNA and adds the indexes and primer sequences for clustering generation. For this library prep, the index adapters used were IDT-ILMN Nextera DNA UD Indexes Set A. After the amplification step, the library was cleaned up using AMPure XP (Beckman Coulter), according to the manufacturer’s protocol. The samples were quantified using Qubit dsDNA HS Assay Kit (Thermo Fisher Scientific) according to the manufacturer’s instructions in the Qubit fluorometer 4 (Thermo Fisher Scientific). The samples were also evaluated on Tapestation 4150 (Agilent) using the kit High Sensitivity D5000 (Agilent), and the average size of the libraries was 284.25. The samples were diluted to 4 nM, and 10 µL of each sample was pooled into a 1.5 mL low-binding tube labeled “library pool.” For denaturation and dilution, the “Standard Normalization Method” from “NextSeq Denature and Dilute Libraries Guide” was performed. Briefly, 0.2 N NaOH was used to denature the 4 nM library pool, followed by dilution with HT1 buffer until the concentration of 1.8 pM (loading concentration). A 1% of PhiX (Illumina) was used as run control. The run was performed using NextSeq 500/550 High Output Kit v2.5 (150 Cycles) reagent kit (Illumina) in the NextSeq 550 sequencing system (Illumina).

### RNA-sequencing analysis

Sample quality was assessed using FASTQC analysis (41). Samples were then trimmed for quality using Cutadapt (42). Paired-end reads were aligned using a custom hg38 human reference genome appended with β-HPV 8 E6 and EGFP genes with the STAR aligner (version 2.7.10b) (43). STAR output files were loaded into featureCounts for quantification (44). Differential expression analysis was performed in R Studio using DESeq2 (45). DESeq2 contrasts were specified for hTERT HFK LXSN samples and for hTERT HFK E6 samples. Gene ontology was performed for genes identified as having reduced expression in hTERT HFK E6 samples as compared to hTERT HFK LXSN samples using ShinyGo (46) and the *clusterProfiler* R package (enrichGO, biological process ontology, Benjamini–Hochberg FDR correction) (47). Innate immune-associated genes were then identified using Innate Immune DB (48). Significant (adjusted *P*-value < 0.05) innate immune-associated genes were extracted from the DESeq2 data set. The contrasts between hTERT HFK LXSN and hTERT HFK E6 samples were then plotted in a heatmap displaying log2 fold change. Expression data for these identified genes were also extracted for each sample, and a variance-stabilizing transformation (VST) was applied. The transformed data were then shown in heatmaps demonstrating fold change of hTERT HFK LXSN and hTERT HFK E6 treated normalized to untreated samples. Normalized counts were extracted from the DESeq2 data set for genes that displayed the largest log2 fold changes. Log fold change (LFC) calculations were then performed using the normalized counts for each biological repeat (*n*=3) and plotted in boxplots. Normalized counts were also extracted for E6 and EGFP, and LFC calculations were performed and displayed using boxplots. A second DESeq2 analysis was run to compare hTERT HFK LXSN- and hTERT HFK E6-untreated samples. After being filtered for significance (adjusted *P*-value < 0.05), DEGs were visualized in a volcano plot. The significant genes were then extracted, and gene ontology was performed using ShinyGO (46).

### Statistical analysis

Statistical significance was determined using Student’s *t*-test. Results were reported as significant for all *P* values <0.05 unless otherwise specified.

## RESULTS

### β-HPV 8 E6 impairs innate immune responses

Given reports that the oncogenes of alpha-HPVs can suppress innate immune response and alter cGAS-STING signaling, we hypothesized that the E6 protein of β-HPV 8 would follow a comparable trend. To examine this, we performed an unbiased assessment of innate immune activation via RNA-seq analysis. Additionally, we examined production of viperin protein, an ISG stimulated by cGAS-STING pathway activation, by immunoblotting.

We performed Illumina RNA-sequencing on hTERT HFKs expressing an empty vector control (hTERT HFK LXSN) and hTERT HFKs expressing HA-tagged HPV 8 E6 driven by the same promoter (hTERT HFK E6) cells 20 h after transfection with 1 µg of pLVX-GFP plasmid. These conditions were chosen because they produced demonstrable phosphorylation of STING and production of viperin in HFK LXSN cells (Fig. S1). Prior to sequencing, transfection success was evaluated via immunoblot (Fig. S2A) and compared to previous immunoblot experiments (Fig. S1). Sequencing was performed using the Illumina NextSeq 550 sequencing system. After sequencing, data quality was analyzed using FASTQC. Reads were then trimmed for quality using Cutadapt and aligned using the STAR aligner. Gene counts were quantified using featureCounts and used to perform differential gene expression (DGE) analysis using DESeq2. E6 expression was used to confirm the validity of hTERT HFK LXSN and hTERT HFK E6 samples, and EGFP expression was evaluated as a marker of transfection success for treated samples (Fig. S1B).

Prior to evaluating the impact of transfections on gene expression, untreated samples were analyzed for differences in gene expression between hTERT HFK LXSN cells and hTERT HFK E6 cells (Fig. S3A). Gene ontology was performed using ShinyGO on significant DEGs. When comparing hTERT HFK E6-untreated cells to hTERT HFK LXSN-untreated cells, genes associated with GO Biological Process terms relating to development and differentiation were found to be downregulated (Fig. S3B). Notably, genes upregulated in hTERT HFK E6 cells were closely related to GO biological process terms relating to innate immunity and cellular responses to viral infection (Fig. S3C). This result shows increased expression of innate immune genes even in the absence of stimulation with dsDNA transfections. Despite the increased expression of cGAS-STING pathway genes, HPV 8 E6 promotes rather than restricts cell growth. This suggests that HPV 8 E6 increases the tolerance of cGAS-STING pathway activation. We therefore compared pathway induction in each cell line relative to its own baseline, representing a level of cGAS-STING activation that does not exert a significant cytostatic effect.

**Fig 1:**
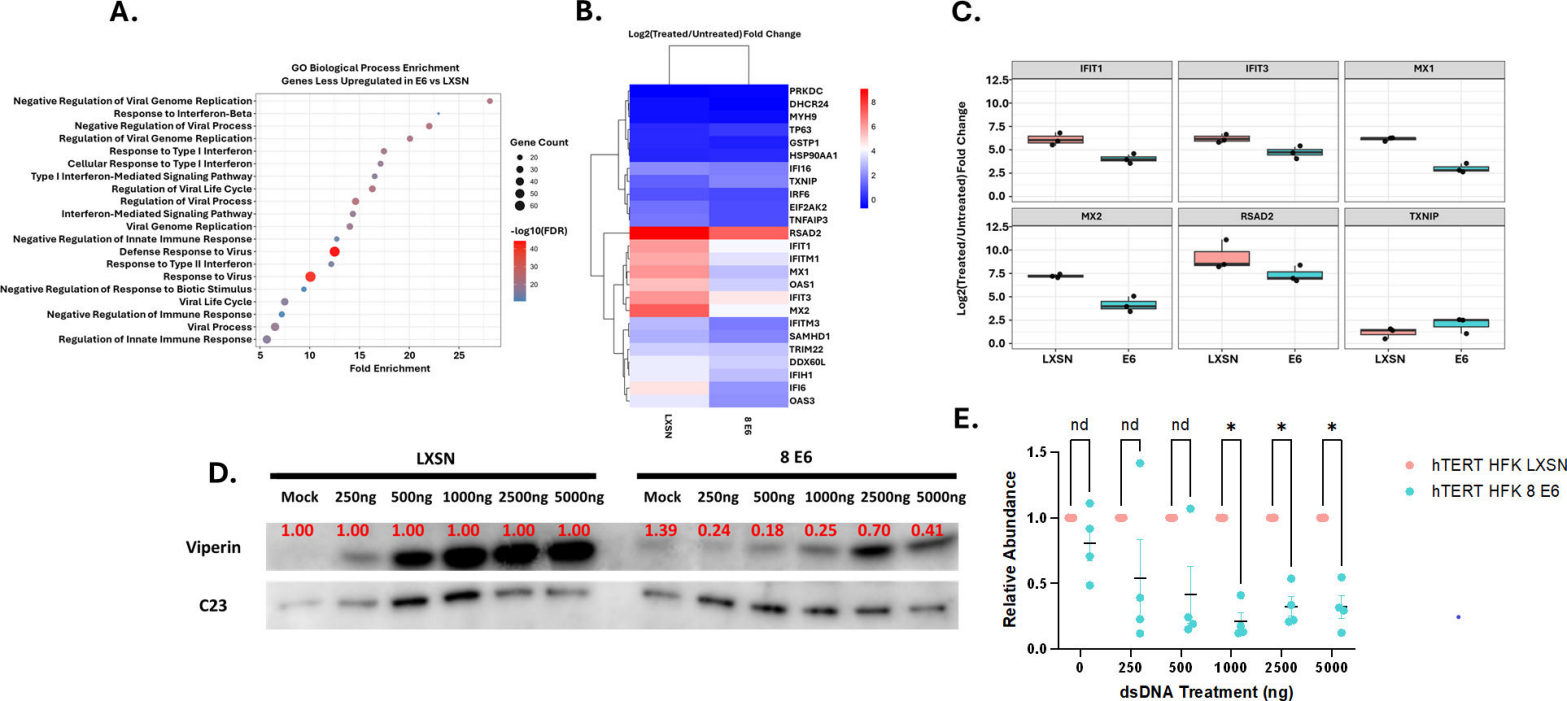
E6 impairs innate immune responses. hTERT HFK LXSN (HFK LXSN) and hTERT HFK E6 (HFK 8 E6) cells were transfected with 1 µg of pLVX-GFP plasmid and harvested 20 h post-transfection. RNA was isolated and purified, then submitted for Illumina sequencing. Differentially expressed genes were identified as having *P*-values less than 0.05 using DESeq2 software. (**A**) Gene ontology analysis of GO biological process terms generated using clusterProfiler from genes identified as less upregulated in response to treatment in hTERT HFK E6 samples as compared to the hTERT HFK LXSN samples. (**B**) Heat map showing log2 fold change of the top 25 significant (adjusted *P*-value < 0.05) differentially expressed innate immune-associated genes between hTERT HFK LXSN-treated and untreated cells and hTERT HFK 8 E6-treated and untreated cells. (**C**) Box plots of the log2 fold change between LXSN and E6-treated and untreated cells for RSAD2 and the top five most variable innate immune genes in response to treatment. LFC calculations were performed using normalized counts extracted for genes showing large differences in expression. Data points represent LFC comparison for each individual biological repeat (*n* = 3). (**D**) Representative western blot demonstrating that the relative abundance of viperin protein is reduced in hTERT HFK 8E6 cells as compared to hTERT HFK LXSN when transfected with 1,000–5,000 ng of pLVX-GFP. (**E**) Quantification of viperin western blots, normalized to the corresponding hTERT HFK LXSN value and NUCLEOLIN (C23).

When evaluating total significant DEGs, an adjusted *P*-value of less than 0.1 was used. When comparing hTERT HFK LXSN-untreated and hTERT HFK LXSN-treated samples, 1,131 DEGs were identified. Of these DEGs, 742 were upregulated, and 389 were downregulated. When comparing hTERT HFK E6-treated and untreated cells, there were 2,237 DEGs. Of these, 1,294 were upregulated, and 943 were downregulated. Both data sets were filtered by adjusted *P*-value of <0.05, and genes that had reduced expression in the hTERT HFK E6 data set compared to the hTERT HFK LXSN data set were extracted. Gene ontology was performed using clusterProfiler (Fig. 1A). Data sets were further filtered to include only innate immune-associated genes that were identified using Innate Immune DB (Fig. 1B). Additional heatmaps were generated for hTERT HFK LXSN and hTERT HFK E6 samples, examining the relative expression of the identified innate immune-associated genes within each cell line (Fig. S4). Here, we demonstrate clear activation of the innate immune response in both cell lines following transfection with dsDNA. However, the induction of innate immune gene expression is substantially lower in hTERT HFK E6 samples than in their LXSN counterparts in many cases. For genes that displayed high levels of expression variance between hTERT HFK LXSN and hTERT HFK E6 samples, normalized counts generated from the DESeq2 data set were used to perform LFC calculations for each biological repeat (*n* = 3) of the experiment (Fig. 3C). Taken together, these data demonstrate that E6 is capable of broadly suppressing innate immune activation in response to stimulation with dsDNA.

### cGAS localizes to β-HPV 8 E6-induced micronuclei

**Fig 2:**
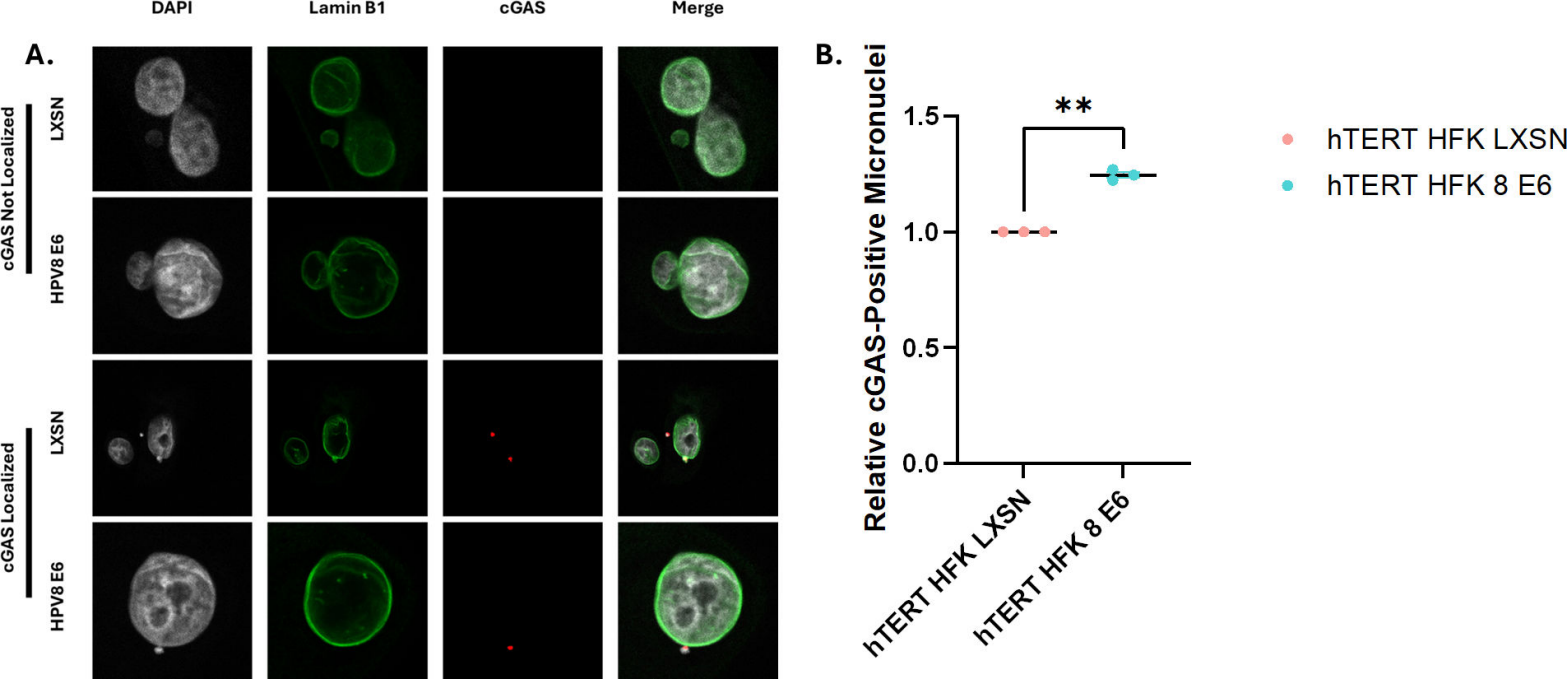
cGAS localizes to micronuclei in hTERT HFK E6 cells. (**A**) Representative immunofluorescent images of cGAS localization to micronuclei in hTERT HFK E6 and LXSN cells. (**B**) Quantification of cGAS-positive micronuclei in hTERT HFK LXSN and hTERT HFK E6 cells. Results were averaged between three independent experiments. At least 250 micronuclei/cell line were quantified for cGAS frequency across the three experiments. The graph depicts ± standard error of the mean. ** *P* < 0.01.

We previously reported that hTERT HFK E6 cells have more micronuclei than hTERT HFK LXSN cells (20). Micronuclei are whole or partial chromosomes existing outside of the primary nucleus (49). Because cGAS-STING signaling is activated in response to micronuclei, we analyzed cGAS activation in response to micronuclei present in hTERT HFK LXSN and hTERT HFK E6 cells (Fig. 2). Even in hTERT HFK E6 cells, micronuclei only occur in a subset of cells, so we could not perform the same detailed immunoblot-driven analysis described above. Instead, we used immunofluorescent microscopy to detect cGAS localization to micronuclei, which serves as an established proxy for activation of the pathway at large (30, 50). Despite the suppression of ISG and viperin production, there was an elevation in the localization of cGAS to micronuclei in hTERT HFK e6 cells (Fig. 2). This demonstrates that the cGAS-STING pathway is initiated in response to β-HPV 8 E6-induced micronuclei. cGAS localization to micronuclei has been linked with chromothripsis, a highly mutagenic process where large stretches of a chromosome experience massive, clustered rearrangements in a single, catastrophic event (51). Thus, these observations are consistent with our previous observation that β-HPV 8 E6 promotes chromothripsis.

### β-HPV 8 E6 reduces phosphorylation of STING in response to double-stranded DNA

**Fig 3:**
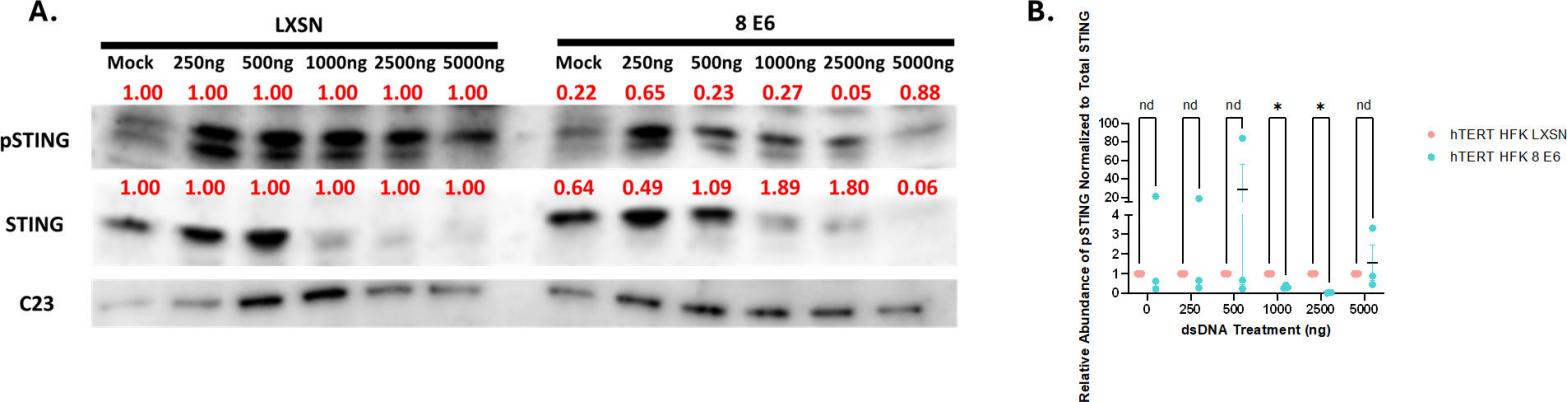
E6 decreases phosphorylation of STING in response to dsDNA. Representative immunoblots of dsDNA-dose response transfected hTERT HFK LXSN (HFK LXSN) and hTERT HFK 8 E6 (HFK 8 E6) cell lines. Immunoblot experiments were repeated in *n* = 3. NUCLEOLIN (C23) was used as a loading control for all immunoblots. Cells were transfected with 0–5 µg of pLVX-GFP plasmid and then harvested 20 h post-transfection. Red numbers above bands show densitometry quantification normalized to HFK LXSN at each treatment dose and NUCLEOLIN loading control. (**A**) Representative immunoblot of pSTING and total STING abundance. The blot displayed here is from the same gel as Fig. 1D and thus has the same loading control. (**B**) Quantification of pSTING abundance after controlling for total STING.

To further characterize E6 suppression of the cGAS-STING pathway, we transfected hTERT HFK LXSN and hTERT HFK E6 cells with a gradient of pLVX-GFP plasmid concentrations (0–5 µg) and harvested protein lysates at 20 h post-transfection. Immunoblot analysis found that these transfections resulted in phosphorylation of STING, indicating cGAS-STING pathway activation in hTERT HFK LXSN and hTERT HFK E6 (Fig. 3). However, this response was significantly more muted in hTERT HFK E6 cells in response to transfection with 1,000 ng of pLVX-GFP.

## DISCUSSION

The ubiquity of β-HPVs suggests that they possess mechanism(s) for evading immune responses. The cGAS-STING innate immune response should effectively suppress β-HPV infections and prevent the genome-destabilizing mutations that arise because of infections. Although β-HPV 8 E6 cells exhibit attenuated viperin production and innate immune gene expression in response to dsDNA treatment (Fig. 1), cGAS localization to micronuclei is increased in these cells (Fig. 2). We therefore hypothesize that the reduced innate immune response results from decreased STING phosphorylation (Fig. 3). Our results provide evidence that STING interference is instead occurring at a post-translational modification level. Expression analysis revealed activation of the innate immune response in both hTERT HFK LXSN and hTERT HFK E6 cells, evidenced by elevated expression of innate immune-related genes. Even without treatment, hTERT HFK E6 cells showed stronger innate immune activation than untreated LXSN controls, indicating that HPV 8 E6 elevates baseline innate immunity. However, this baseline induction is insufficient to suppress proliferation. To account for these baseline differences, gene expression levels were compared between E6 and LXSN cells after normalization to their respective untreated states. Following dsDNA transfection, hTERT HFK E6 cells mounted an overall attenuated response compared to LXSN cells, with reduced induction of the expression of multiple innate immune, antiviral, and other genes associated with immune response-related GO biological processes (Fig. 1A through C). Many of the observed differentially expressed genes are interferon-inducible proteins that have key roles in modulating the antiviral innate immune response. Many α-HPVs display a similar ability to downregulate these same antiviral genes, suggesting a shared need to attenuate the innate immune system (35, 36, 38, 39, 52). The extent to which innate immune suppression is shared among other papillomaviruses represents an area for future examination.

Our group and others have shown that β-HPV 8 E6 promotes host cell growth in the face of diverse anti-proliferative stimuli. For example, β-HPV 8 E6 alters DNA repair responses and promotes growth despite failed cytokinesis (20, 53–63). This likely stems from the known requirement of proliferating cells for papillomavirus replication (64). While these pro-proliferative activities likely facilitate β-HPV 8 propagation, they are expected to also induce genomic instability through the introduction of micronuclei, mutations, and large-scale genomic rearrangements via chromothripsis (53, 58). The ability to attenuate innate immune responses described here is expected to have similar advantages for the virus and consequences for the host. While avoiding innate immune activation would have obvious benefits for viral propagation, innate immunity is critical for safeguarding genome stability by eliminating cells with DNA in their cytoplasm, where it is at high risk for mutations.

While this work highlights the ability of β-HPV 8 E6 to impair the cGAS-STING innate immune response, there are still questions as to what extent β-HPVs impair innate immunity. Likewise, our work focuses on E6 protein isolation, leaving it unclear how the expression of E6 in the context of the complete early region would augment or suppress its impact on cGAS-STING signaling. The published literature suggests that the complete early region is more capable of suppressing innate immune signaling. Rattay et al. (65) identified roles for β-HPV 8 E1 and E2 genes to antagonize PRRs in cells. There are also several documented roles for α-HPV E7 in impairing cGAS-STING signaling (26, 66). We have yet to determine the mechanism for pathway impairment following STING phosphorylation. Finally, while other papers have examined β-HPV effect on RIG-1 and TLR signaling (65, 67), the full impairment of the innate immune response has yet to be characterized at a transcriptional and post-translational modification level.

## SUPPLEMENTAL MATERIAL

Supplemental Figures msphere.00859-25-s0001.pdf Fig. S1 to S4.

ASM does not own the copyrights to Supplemental Material that may be linked to, or accessed through, an article. The authors have granted ASM a non-exclusive, world-wide license to publish the Supplemental Material files. Please contact the corresponding author directly for reuse.
