# Supplementary figures and images for: Cutaneous human papillomavirus E6 impairs the cGAS-STING pathway
Source: mSphere. 2026 Feb 26;11(3):e00859-25. doi: 10.1128/msphere.00859-25 (PMC13037410; doi:10.1128/msphere.00859-25)

# Supplemental Figures

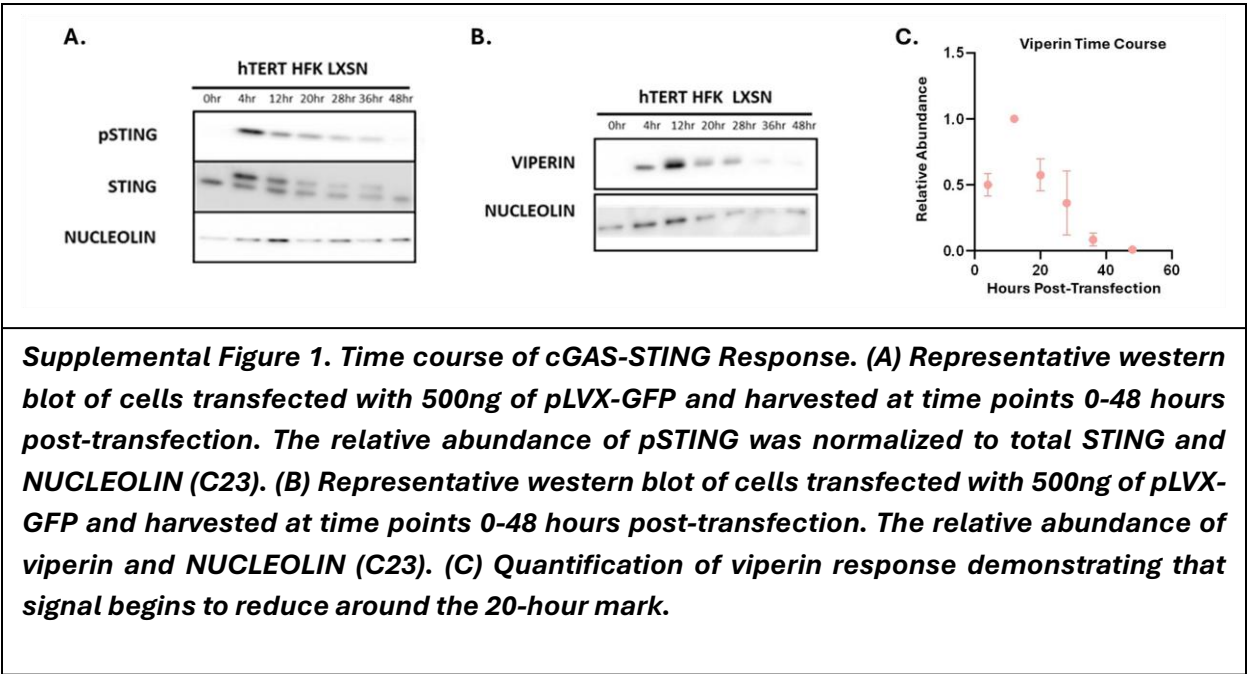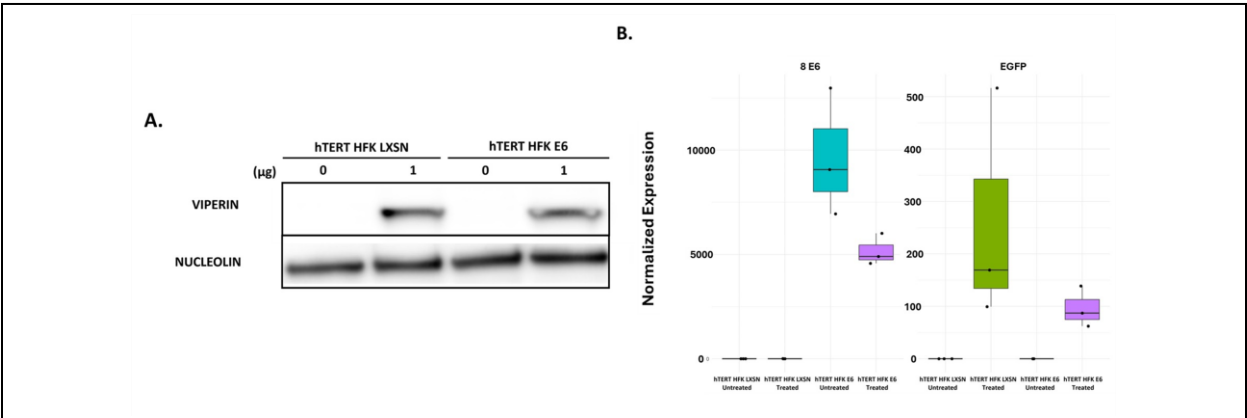

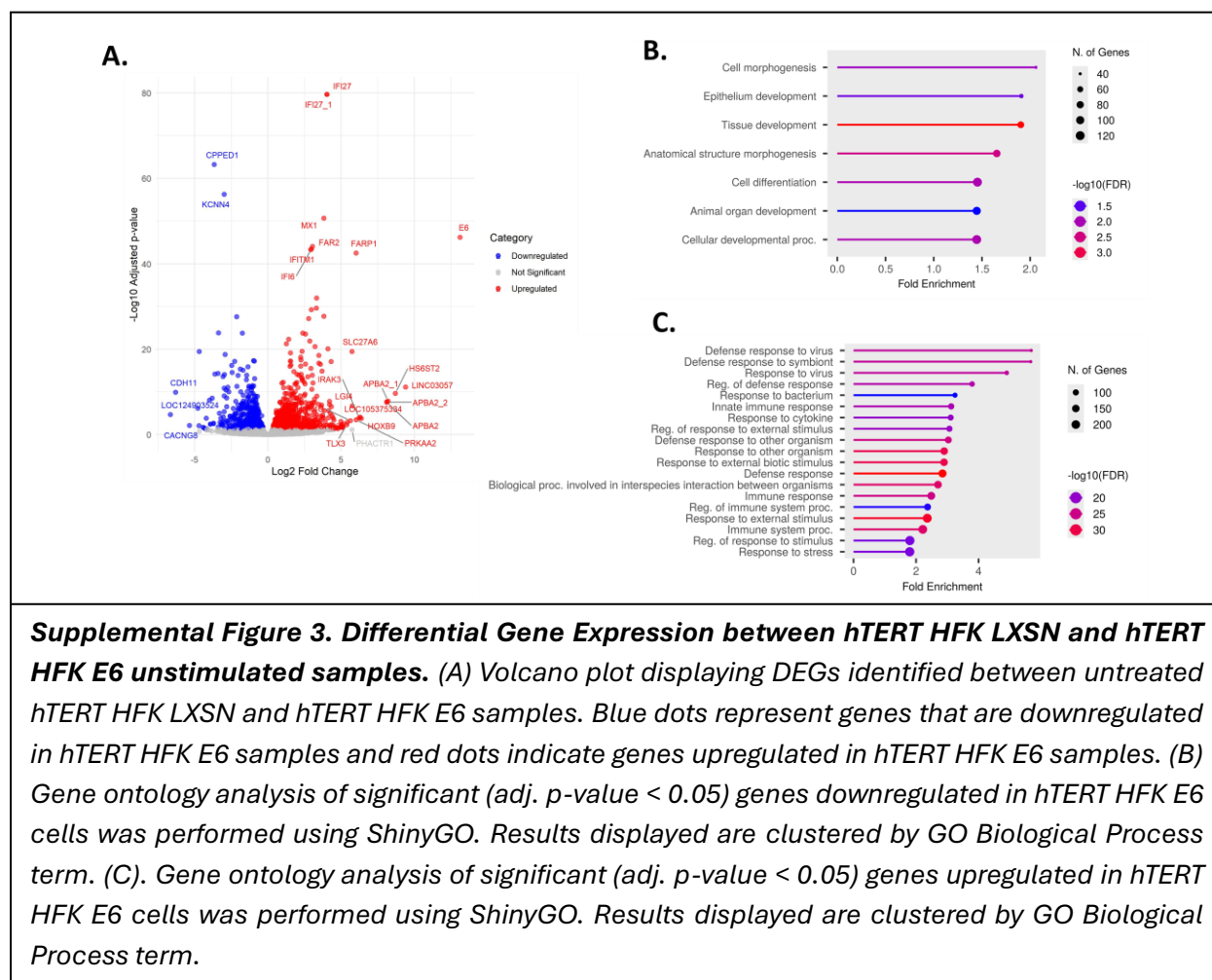

Supplement: Supplemental Figures — Fig. S1 to S4. [file msphere.00859-25-s0001.pdf]
